# Research Progress of circRNAs in Head and Neck Cancers

**DOI:** 10.3389/fonc.2021.616202

**Published:** 2021-04-29

**Authors:** Panchun Li, Kunjie Zhu, Yongzhen Mo, Xiangying Deng, Xianjie Jiang, Lei Shi, Can Guo, Wenling Zhang, Zhaoyang Zeng, Guiyuan Li, Wei Xiong, Shanshan Zhang, Zhaojian Gong

**Affiliations:** ^1^ Department of Oral and Maxillofacial Surgery, The Second Xiangya Hospital, Central South University, Changsha, China; ^2^ Department of Head and Neck Surgery, The Affiliated Cancer Hospital of Xiangya School of Medicine, Central South University, Changsha, China; ^3^ NHC Key Laboratory of Carcinogenesis, Cancer Research Institute and School of Basic Medical Sciences, Central South University, Changsha, China; ^4^ Department of Pathology, The Second Xiangya Hospital, Central South University, Changsha, China; ^5^ Department of Stomatology, The Key Laboratory of Carcinogenesis and Cancer Invasion of the Chinese Ministry of Education, Xiangya Hospital, Central South University, Changsha, China

**Keywords:** circRNA, non-coding RNA, biomarker, head and neck cancer, miRNA sponge

## Abstract

Circular RNAs (circRNAs) are a novel type of non-coding RNAs. Because of their characteristics of a closed loop structure, disease- and tissue-specificity, and high conservation and stability, circRNAs have the potential to be biomarkers for disease diagnosis. Head and neck cancers are one of the most common malignant tumors with high incidence rates globally. Affected patients are often diagnosed at the advanced stage with poor prognosis, owing to the concealment of anatomic sites. The characteristics, functions, and specific mechanisms of circRNAs in head and neck cancers are increasingly being discovered, and they have important clinical significance for the early diagnosis, treatment, and prognosis evaluation of patients with cancer. In this study, the generation, characteristics, and functions of circRNAs, along with their regulatory mechanisms in head and neck cancers have been summarized. We report that circRNAs interact with molecules such as transcription and growth factors to influence specific pathways involved in tumorigenesis. We conclude that circRNAs have an important role to play in the proliferation, invasion, metastasis, energy and substance metabolism, and treatment resistance in cancers.

## Introduction

Head and neck cancers (HNC) are malignant tumors occurring in the oral cavity, hypopharynx, nasopharynx, and larynx. Depending on the location, HNC can be divided into hypopharyngeal squamous cell carcinoma (HSCC), oral squamous cell carcinoma (OSCC), laryngeal squamous cell carcinoma (LSCC), and nasopharyngeal carcinoma (NPC). With about 600,000 new patients per year, HNC ranks seventh in the incidence rate of malignant tumors worldwide (1). There are many ways to treat HNC, such as surgical treatment, radiotherapy, and chemotherapy. However, due to the lack of early diagnosis methods, patients with HNC are often diagnosed in the late stage. The 5-year overall survival rate of patients is 54.7%–65.9% and has not significantly improved over the years (2, 3). Therefore, it is necessary to elucidate the potential molecular regulation mechanism of HNC and to find possible molecular markers and targets. Circular RNAs (circRNAs) have the potential to be HNC biomarkers (4). This review summarizes the research progress of circRNAs in HNC and seeks to understand the occurrence, development, and metastasis of HNC, and the possible molecular markers and therapeutic targets.

## Classification and Formation of circRNA

According to the composition of the source, circRNA can be divided into 4 categories: exonic circRNAs (ecircRNA), intronic circRNAs (ciRNAs), exon-intron circRNAs (EIciRNA), and tRNA intronic circRNA(tricRNA) (5). Although the formation mechanism of circRNAs is not completely clear, the current research results reveal two different formation modes (4).

Lariat-driven circularization: Lasso-driven cyclization is also known as the exon-skipping model (4), whereby non-adjacent exons of pre-mRNA are close to each other during transcription, causing part of exons to be skipped and eventually not appear on mature mRNA. By exon skipping, the exons and introns that are skipped form a lasso structure, and after the introns are removed, the ecircRNA is formed.

Intron-pairing-driven circularization model: There are reverse complementary sequences in flanking introns of pre-mRNA, which can assist pre-mRNA to form lasso structures through complementary base pairing. For example, inverted repeated Alu sequences can make introns move close to each other in space and form a hairpin structure, and finally form a lasso structure containing exons and introns. After the introns are removed, ecircRNA containing only the exons is formed (6). During this process, introns are sometimes retained in the circRNA resulting in formation of EIciRNA (7). However, the specific mechanisms of EIciRNA formation are not clear. In addition, when the two ends of an intron have complementary sequences, the cyclization of a single intron can occur and form ciRNA (8). The formation of ciRNA (mainly occurring in the nucleus) depends on a consensus motif containing a seven-nucleotide (nt) GU-rich element near the 5′ splice site and an 11 nt C-rich element close to the branchpoint site (8).

RNA-binding proteins (RBP) are also involved in the circRNA cyclization; they can bind to introns, assist the interaction of upstream and downstream introns, and promote circRNA cyclization (8). Quaking is an RBP, which can form dimers and bind to the binding site of flanking introns, and promote the production of circRNA (9).

## Characteristics of circRNA

Up to now, more than one million circRNAs have been found in human tissues by high-throughput sequencing technology. Because of their characteristics of a closed loop structure, disease- and tissue-specificity, and high conservation and stability, circRNAs have the potential to be biomarkers for disease diagnosis.

The highly stable structure of circRNAs is a closed loop, without 3′ and 5′ ends and a poly (A) tail. Therefore, compared with linear RNAs, circRNAs are not easily hydrolyzed by RNase. It is reported that the half-life of circRNAs can reach more than 48 h, while that of linear RNAs is only 10 h (10).

Types and expression abundance: circRNAs are numerous and widely available in human cells (11). Over 1,000,000 circRNAs have been found in human tissues using high-throughput sequencing technology (4). The expression abundance of different circRNAs varies greatly. The expression of some circRNAs is much higher than that of linear mRNAs, and even more than 10 times that of their linear isomers (4). However, most circRNAs have low expression abundance.

Conservation: Because of the stability of circRNAs, most of them are conserved in the evolution process of different species (12, 13). For example, 5¬–30% of circRNAs from human and mouse homologous genes are completely conserved (14). The conservation of circRNAs proves that they are not by-products of transcription but play an important role in life activities.

Expression specificity: The expression of circRNAs is tissue- and disease-specific (12). The expression of circRNAs in embryo, brain, heart, stomach, lung, liver, and other organs is very different. For example, in the brain, the amount of circRNAs is higher than that of other tissues (15, 16). Expression specificity indicates that circRNAs may play a specific function in disease development and may serve as a biomarker for disease diagnosis.

## The Functions of circRNAs

miRNA sponging: CircRNAs are rich in miRNA binding sites, which can act as competitive endogenous RNA (ceRNA) to adsorb miRNA, thereby regulating the activity of downstream target genes of miRNA (11). For example, CDR1as contains more than 70 miR-7 binding sites, which act as ceRNA regulatory target genes in many biological processes (16–19).

Combination with protein: CircRNAs can directly bind to proteins and affect the function of downstream proteins. For example, circ-CCND1 can inhibit the binding of HuR to CCND1 mRNA by binding to HUR (20). In addition, circRNAs can act as a scaffold to make multiple proteins form complex and accelerate the reaction.

Translation of proteins: CircRNAs without 5’cap and 3’poly(A) tail lack internal ribosome entry site, so circRNAs are defined as non-coding RNA. However, studies have shown that circRNAs with open reading frames can translate functional proteins through internal ribosome entry site (IRES) or m6A-mediated translation initiation mechanisms (21). IRES can fold into a tRNA-like structure and mediate the binding of ribosomes to RNA. For example, circAKT3 in brain tumors can encode functional protein AKT3-174aa through IRES, and regulate PI3K/AKT signaling pathway (22). In addition, m6A modified circRNAs can directly bind to eukaryotic initiation factor 3, realizing a cap-independent translation initiation mode (23).

## CircRNAs Are Widely Expressed and Serve as Markers and Targets in HNC

With the development of high-throughput sequencing, microarray technology, and bioinformatics, it has been increasingly found that circRNAs have abnormal expression and potential functions in HNC (24–27). For example, in HSCC, Feng et al. analyzed the expression profiles of four paired HSCC tissues and found 173 differentially expressed circRNAs. The authors performed enrichment analysis on 40 circRNAs with the most significant differences and found that these circRNAs are most likely to play a role in the ErbB and Hippo signaling pathways (28). In addition, Cheng et al. constructed a circRNA expression profile of LSCC by performing second generation sequencing on five paired LSCC tissues. They detected 21,444 circRNAs, and further analysis using the Kyoto Encyclopedia of Genes and Genomes showed that peroxisome proliferator-activated receptor-, axon guidance, and Wnt signaling pathways are the key pathways of circRNAs in the occurrence of LSCC (26).

CircRNAs are not only widely expressed in HNC but are also correlated to the prognosis of patients. They have the potential as biomarkers for tumor diagnosis and prognosis (29–37). For example, the expression of hsa_circ_0092125 is related to the tumor size, tumor node metastasis stage, and lymph node metastasis in OSCC patients. Survival analysis shows that patients with a higher expression of hsa_circ_0092125 survive longer (29).

The receiver operating characteristic (ROC) curve and the area under curve (AUC) can be used as indices to evaluate the accuracy of biomarkers. The AUC values of hsa_circ_0072387, hsa_circ_001242, hsa_circ_009755, hsa_circ_0004491, hsa_circ_0036722, hsa_circ_0008309 and hsa_circ_0086414 are 0.746, 0.784, 0.782, 0.751, 0.838, 0.764 and 0.749, indicating the reliability of these molecules as biomarkers (30–37). Body fluids also contain a large amount of circRNAs. For example, Zhao et al. reported that the expression of hsa_circ_0001874 and hsa_circ_0001971 was upregulated in OSCC saliva, and the AUC value of the combination of these two molecules was 0.922 (33). Serum exosomes can reflect the pathological conditions of the body and are considered as a potential auxiliary diagnostic marker. CircRASSF2 is upregulated in the serum exosomes of patients with LSCC and can affect the progression of LSCC (38). Those circRNAs in body fluids can be easily detected and have a great clinical value and significance.

In addition, circRNAs have the potential to be therapeutic targets. Fucoidan is a component of the seaweed cell wall, which can inhibit OSCC growth, migration, and invasion. Zhang et al. reported that circFLNA is a fucoidan target. They found that fucoidan increased the expression of circFLNA in OSCC cell lines and affected the expression of key proteins related to growth, apoptosis, migration, and invasion (39).

## CircRNAs Are Associated With Proliferation in HNC

**Figure 1:**
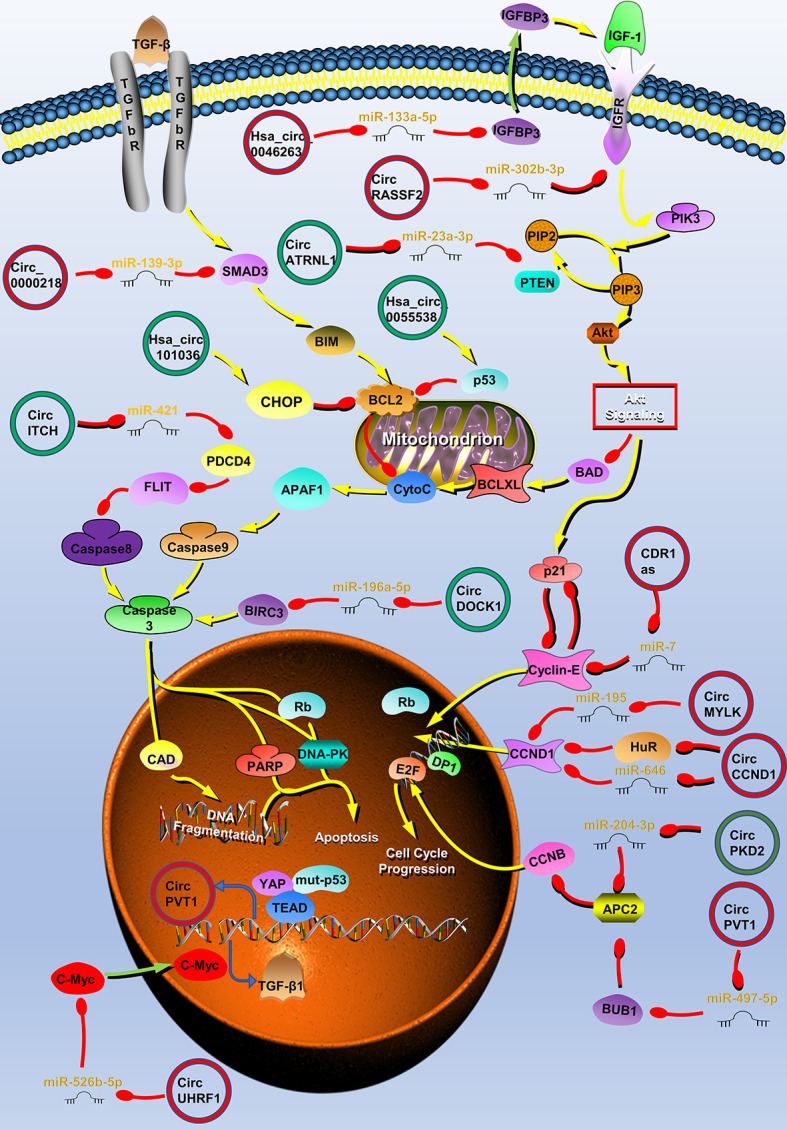
Circular RNAs (circRNAs) associated with proliferation in head and neck cancers (HNC). CircRNAs that are known to regulate proliferation processes and their validated targets in HNC. CircRNAs regulate the growth of HNC by regulating PI3K/Akt/mTOR, insulin-like growth factor (IGFR), transforming growth factor (TGF) β, p53 signaling pathways, apoptosis, and cell cycle-related molecules. Red circle, upregulated circRNAs in HNC; green circle, downregulated circRNAs in HNC; yellow arrow, positive regulation; red arrow, negative regulation.

The uncontrolled proliferation of tumor cells is the basis of tumorigenesis. Multiple circRNAs have been demonstrated to be involved in HNC development through the regulation of the proliferation process (**Figure 1**). CircATRNL1 (40), hsa_circ_0007059 (41), circRNA_002178 (42), hsa_circ_0046263 (43), hsa_circ_0005379 (44), circUHRF1 (45), circPVT1 (46, 47), circHIPK3 (48, 49), circRNA_0000140 (50), circRNA_100290 (51), circDOCK1 (52), hsa_circ_0055538 (53), circITCH (54), hsa_circRNA_101036 (55), circCCND1 (20), circMYLK (56), CDR1as (17, 57, 58), circPKD2 (59), circLARP4 (60), hsa_circ_0042666 (61), hsa_circRNA_100533 (62), circMDM2 (63), circ_0067934 (64), circRPMS1 (65), circ-ZNF609 (66, 67), hsa_circ_0072387 (68), circRASSF2 (38), hsa_circ_0023028 (69), circCTDP1 (70), circ_0008450 (71), circMATR3 (72), hsa_circ_0057481 (73), circ_0001742 (74), circ_0000218 (75), circ_0001971 (76), circCORO1C (77), hsa_circ_0109291 (78), hsa_circ_0036722 (34), hsa_circ_0002203 (79) and hsa_circ_0066755 (80) are associated with tumor proliferation (**Table 1**).

**Table 1:** The proliferation-related circular RNAs in head and neck cancers.

CircRNAs	Primary sites of cancer	Expression change	Mechanism	Functions	References
CircPVT1	HNC	Up	mut-p53-YAP-TEAD complex/ CircPVT1/miR-497-5p/bub1	Promote proliferation	(46)
CircPVT1	OSCC	Up	miR−125b/STAT3	Promote proliferation	(47)
CDR1as	NPC	Up	miR-7-5p/E2F3	Promote proliferation	(57)
CDR1as	LSCC	Up	miR-7/CCNE1 and PIK3CD	Promote proliferation	(17)
CDR1as	OSCC	Up	miR-671-5p and AKT/ERK/mTOR	Promote proliferation	(58)
Circ-ZNF609	NPC	Up	microRNA-150-5p/Sp1	Promote proliferation	(66)
Circ-ZNF609	NPC	Up	miR-188/ELF2	Promote proliferation	(67)
CircHIPK3	NPC	Up	miR-4288/ELF3	Promote proliferation	(48)
CircHIPK3	OSCC	Up	miR-124	Promote proliferation	(49)
CircMDM2	OSCC	Up	miR-532-3p/HK2	Promote proliferation	(63)
Circ_0001971	OSCC	Up	miR-194 and miR-204	Promote proliferation	(33)
Circ_0001742	OSCC	Up	miR-431-5p/ATF3、miR-634/RAB1A	Promote proliferation	(74)
Circ_0000218	LSCC	Up	miR-139-3p/Smad3	Promote proliferation	(75)
CircUHRF1	OSCC	Up	miR-526b-5p/c-Myc/TGF-β1/ESRP1feedback loop	Promote proliferation	(45)
Hsa_circ_0072387	OSCC	Up	–	Promote proliferation	(68)
Hsa_circ_0109291	OSCC	Up	–	Promote proliferation	(78)
CircRPMS1	NPC	Up	miR-203, miR-31 and miR-451	Promote proliferation	(65)
CircCTDP1	NPC	Up	microRNA-320b/HOXA10/TGFβ2	Promote proliferation	(70)
Circ_0008450	NPC	Up	miR-577/CXCL9	Promote proliferation	(71)
Hsa_circ_0046263	NPC	Up	miR-133a-5p/IGFBP3	Promote proliferation	(43)
Circ_0067934	LSCC	Up	miR-1324	Promote proliferation	(64)
Circ-CCND1	LSCC	Up	HuR and miR-646/CCND1	Promote proliferation	(20)
CircMYLK	LSCC	Up	miR-195/CCND1	Promote proliferation	(56)
CircRASSF2	LSCC	Up	miR-302b-3p/IGF-1R	Promote proliferation	(38)
Hsa_circ_0023028	LSCC	Up	miR-194-5p	Promote proliferation	(69)
CircRNA_100290	LSCC	Up	miR-136-5p/RAP2C	Promote proliferation	(51)
Hsa_circRNA_101036	OSCC	Up	CHOP and Bcl-2	Promote proliferation	(55)
Hsa_circ_0057481	LSCC	Up	miR-200c/ZEB1	Promote proliferation	(73)
CircCORO1C	LSCC	Up	let-7c-5p/PBX3	Promote proliferation	(77)
CircMATR3	HSCC	Up	–	Promote proliferation	(72)
CircRNA_002178	OSCC	Up	Akt/mTOR	Promote proliferation	(42)
Hsa_circ_0066755	NPC	Up	miR-651	Promote proliferation	(80)
Hsa_circ_0005379	OSCC	Down	EGFR	Inhibit proliferation	(44)
CircATRNL1	OSCC	Down	miR-23a-3p/PTEN/PI3K/Akt	Inhibit proliferation	(40)
Hsa_CircRNA_100533	OSCC	Down	miR-933/GNAS	Inhibit proliferation	(62)
Hsa_circ_0007059	OSCC	Down	AKT/mTOR	Inhibit proliferation	(41)
Hsa_circ_0055538	OSCC	Down	p53/Bcl-2/caspase	Inhibit proliferation	(53)
Circ-PKD2	OSCC	Down	miR-204-3p/APC2	Inhibit proliferation	(81)
Circ-ITCH	OSCC	Down	miR-421/PDCD4	Inhibit proliferation	(54)
Hsa_circ_0002203	OSCC	Down	–	Inhibit proliferation	(79)
CircLARP4	NPC	Down	ROCK1	Inhibit proliferation	(60)
CircRNA_0000140	OSCC	Down	miR-31/LATS2	Inhibit proliferation	(50)
Hsa_circ_0042666	LSCC	Down	miR-223/TGFBR3	Inhibit proliferation	(61)
Hsa_circ_0036722	LSCC	Down	miR-1248/RHCG	Inhibit proliferation	(34)
circDOCK1	OSCC	Down	miR−196a−5p/BIRC3	Inhibit proliferation	(52)

Tumor proliferation is regulated by multiple cellular pathways, such as the PI3K/Akt/mTOR signaling pathway, and some circRNAs are involved in the regulation of tumor proliferation through these pathways. In OSCC, circATRNL1 can induce apoptosis and cell cycle arrest through the PI3K/Akt/mTOR signaling pathway (40). CircATRNL1 is downregulated in OSCC and can target miR-23a-3p to eliminate the inhibition of phosphatase and tensin homolog (PTEN) by miR-23a-3p. PTEN is one of the most common tumor suppressor genes, which regulates cell apoptosis and cell cycle through PI3K/Akt/mTOR signaling pathway (82). PTEN can enhance BAD activity, inhibit caspase-3 activation, and induce apoptosis by antagonizing the PI3K/Akt/mTOR signaling pathway (83, 84). In addition, PTEN induces the expression of the cell cycle inhibitor p21, resulting in cell G0/G1 arrest (85). Therefore, circATRNL1 affects the progression of OSCC through the PTEN/PI3K/Akt/mTOR signaling pathway (40). Similarly, hsa_circ_0007059 and circRNA_002178 also affect OSCC growth through AKT signaling pathway (41, 42). The transforming growth factor β (TGF-β) signaling pathway can also regulate cell proliferation (86). Zhao et al. reported that circUHRF1 positively regulates the c-Myc oncogene by competitively binding miR-526b-5. In addition to activating the TGF-β1 pathway, c-Myc can also promote the expression of ESRP1. ESRP1 promotes the generation of circUHRF1 by combining with the flanking introns of circUHRF1, and finally forms a positive feedback regulation loop. CircUHRF1 eventually activates the TGF-β pathway to promote tumor growth (45).

Various growth factors such as insulin-like growth factors (IGFs) and their receptors are also involved in the growth of HNC (87, 88). When IGF-1R is combined with its ligand, it can activate the PI3K/Akt/mTOR signaling pathway, and play an important role in the proliferation and apoptosis of tumor cells (88, 89). CircRASSF2 can adsorb miR-302b-3p as a competitive endogenous RNA (ceRNA), increase the expression of IGF-1R, and promote the progression of LSCC (38). IGFBP3 is the transporter of IGF-1, and it can extend the half-life of IGF-1 and promote the binding of IGF-1 and IGF-1R. IGFBP3 is the downstream target of miR-133a-5p and hsa_circ_0046263 acts as a sponge of miR-133a-5p in NPC and promotes IGFBP3 expression. Therefore, hsa_circ_0046263 can also activate the IGFR signaling pathway to promote LSCC (43).

P53 is the most commonly mutated gene in HNC; the mutation and deletion of this gene leads to the disorder of intracellular signal pathways and the uncontrolled growth of tumor cells (90). The expression of circPVT1 is upregulated in patients with HNC with a p53 mutation. Further research found that the complex formed by mutant p53, yes-associated protein, and TEA domain combined with the circPVT1 promoter to regulate circPVT1 expression at the transcriptional level. CircPVT1 acts as an oncogene and can regulate the expression of miR-497-5p and its downstream genes, which are involved in cell proliferation (46). In addition to the above mechanisms, circRNAs can regulate the proliferation of HNC through different mechanisms such as affecting apoptosis and cell cycle.

Resistance to cell death is one of the ten characteristics of tumors. Apoptosis is the main way to clear cells that are potentially harmful to the body, and almost all cancer cells have anti-apoptotic mechanisms (91). CircRNAs can directly regulate molecules related to the apoptosis pathway. For example, Wang et al. found that circDOCK1 can act as the ceRNA of miR−196a−5p to regulate the expression of Baculoviral IAP repeat-containing protein 3 (BIRC3). BIRC3 is an endogenous inhibitor of a cysteine proteolytic enzyme (caspase), which can prevent the conversion of the p19 subunit of caspase-3 to the p17 subunit and inhibit the activity of caspase 3 (92, 93). Wang et al. used tumor necrosis factor α to stimulate the OSCC cell line CAL-27 to establish an OSCC apoptosis model, establish an apoptosis-related circRNA profile through microarray sequencing, and screen the apoptosis-related molecule circDOCK1 (52). Finally, the authors found that circDOCK1 regulates the expression of BIRC3 by acting as a ceRNA and participates in the process of OSCC cell apoptosis (52). The p53 molecule is the central mediating switch of apoptosis and can downregulate the expression of Bcl-2 to promote cell apoptosis (94). Su et al. found that the overexpression of hsa_circ_0055538 in OSCC increased the expression levels of p53, Bax, and caspase 3, and decreased the expression of Bcl-2. These indicate that hsa_circ_0055538 may affect OSCC apoptosis through the p53/Bcl-2/caspase signaling pathway (53). Programmed cell death 4 (PDCD4) is a tumor suppressor gene related to apoptosis. Studies have found that PDCD4 inhibits FLIP-mediated tumor cell apoptosis. In addition, circ-ITCH can directly bind to miR-421 and block its inhibition of PDCD4 in OSCC and induce cell apoptosis (54). Endoplasmic reticulum stress is another way to induce apoptosis. The main signs of endoplasmic reticulum stress are the upregulation of related proteins CHOP and Bcl-2 (95). After the overexpression of hsa_circRNA_101036 in OSCC, the levels of these proteins levels of CHOP and Bcl-2 can be found to increase, and hsa_circRNA_101036 may exert a tumor suppressor effect by regulating the endoplasmic reticulum stress in cancer cells (55). Autophagy is also a way of cell death, but under severe conditions, tumor cells can promote cell survival through autophagy. According to reports, CDR1as can promote autophagy and growth of OSCC by adsorbing miR-671-5p and promoting the AKT/ERK/mTOR signaling pathway (58).

The cell cycle is closely related to the growth of tumors (96). Abnormal expression of cyclin will result in abnormal cell growth. Cyclin D (CCND1) plays an important positive regulatory role in G1/S phase transition. Its high expression can shorten the G1 phase and accelerate the cell cycle. CircCCND1 is a circRNA derived from the CCND1 gene (97). CircCCND1 can block the inhibition of CCND1 by adsorbing miR-646. In addition, circ-CCND1 also inhibits the binding of the human antigen R to CCND1 mRNA by binding to HUR, thereby enhancing the stability of CCND1 mRNA (20). Briefly, circ-CCND1 regulates CCND1 in these two ways and regulates the transformation of cells from G1 to S phase. circMYLK also promotes CCND1 expression in LSCC by binding to miR-195 (56). Cyclin E1 also plays an important role in the G1/S phase transition (98). As the first reported circRNA molecule, CDR1as can inhibit miR-7, upregulate the expression of cyclin E1, and promote LSCC progression (17). Gao et al. found that the expression of circPKD2 was downregulated in the OSCC circRNA expression profile and experimentally proved that circPKD2 can induce cell cycle arrest. CircPKD2 also acts as a sponge for miR-204-3p, inhibiting miR-204-3p from acting on APC2 (59). APC2 can work with DNA-PKcs to inhibit the stability of CCND1 (81). Therefore, circ-PKD2 plays a tumor suppressor effect by promoting APC2.

## CircRNAs Are Associated With Invasion and Metastasis in HNC

**Figure 2:**
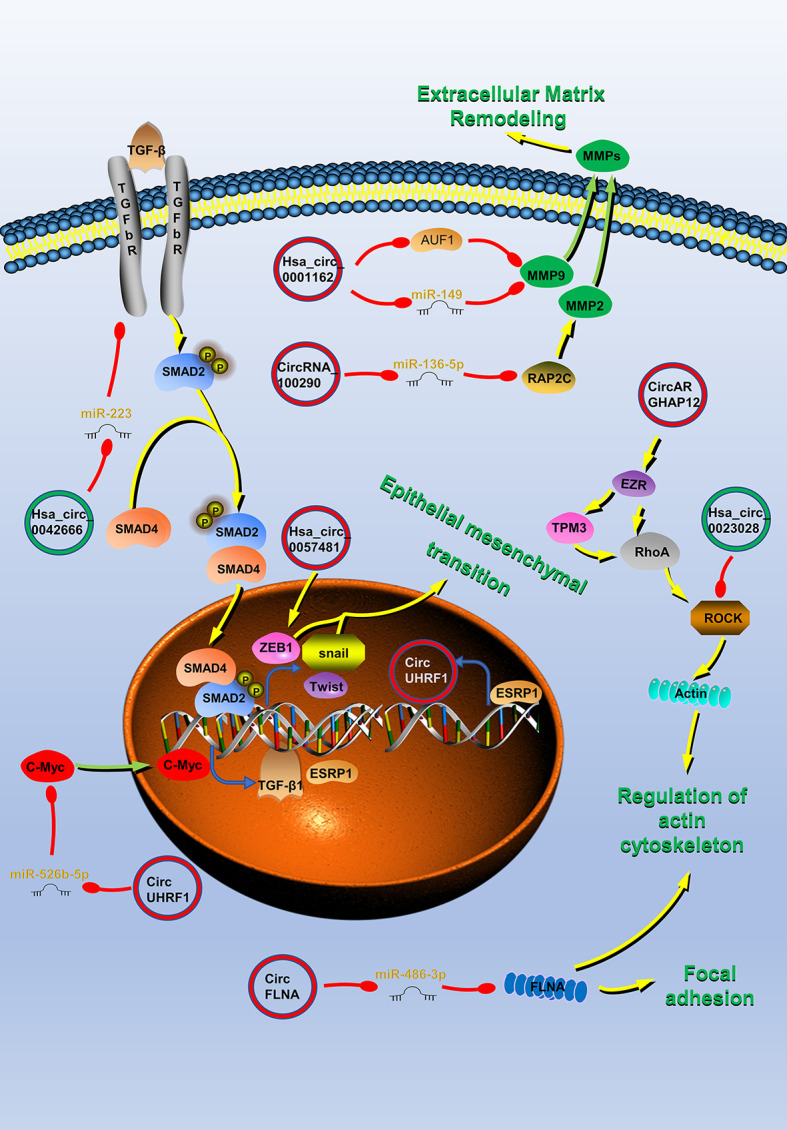
Circular RNAs (circRNAs) associated with invasion and metastasis in head and neck cancers (HNC). CircRNAs that are known to regulate invasion and metastasis processes and their validated targets in HNC. CircRNAs regulate the cytoskeleton remodeling, local adhesion, extracellular matrix remodeling, and the epithelial–mesenchymal transition (EMT) of HNC by regulating invasion and metastasis-related molecules to promote tumor invasion and metastasis. Red circle, upregulated circRNAs in HNC; green circle, downregulated circRNAs in HNC; yellow arrow, positive regulation; red arrow, negative regulation.

Invasion and metastasis are the most essential biological features of malignant tumors and are also the direct cause of treatment failure in tumor patients. CircRNAs in HNC have important roles in HNC metastasis and invasion (**Figure 2**). Hsa_circ_0007059 (41), circUHRF1 (45), circHIPK3 (48), circRNA_0000140 (50), circRNA_100290 (51), hsa_circ_0055538 (53), CDR1as (17, 57), circPKD2 (59), circLARP4 (60), hsa_circ_0042666 (61), hsa_circRNA_100533 (62), circ_0067934 (64), circRPMS1 (65), circ-ZNF609 (66), hsa_circ_0072387 (68), circRASSF2 (38), hsa_circ_0023028 (69), circCTDP1 (70), circ_0008450 (71), circMATR3 (72), hsa_circ_0057481 (73), circ_0001742 (74), circCORO1C (77), hsa_circ_0004491 (37), circ_0001971 (33), circFLNA (99), hsa_circ_0001162 (100), circARHGAP12 (101), circCRIM1 (102), circEPSTI1 (103), hsa_circ_0109291 (78), circRNA_002178 (42), hsa_circ_0002203 (79) and hsa_circ_0066755 (80) are involved in the regulation of HNC invasion and metastasis (**Table 2**).

**Table 2:** The migration and invasion-related circular RNAs in head and neck cancers.

CircRNAs	Primary sites of cancer	Expression change	Mechanism	Functions	References
Circ_0001971	OSCC	Up	miR-194 and miR-204	Promote invasion and metastasis	(33)
Hsa_circ_0001162	OSCC	Up	AUF1 and miR-149/MMP9	Promote invasion and metastasis	(100)
CircUHRF1	OSCC	Up	miR-526b-5p/c-Myc/TGF-β1/ ESRP1feedback loop	Promote invasion and metastasis	(45)
CircEPSTI1	OSCC	Up	mir-942-5p/LTBP2	Promote invasion and metastasis	(103)
Circ_0001742	OSCC	Up	miR-431-5p/ATF3、miR-634/RAB1A	Promote invasion and metastasis	(74)
Hsa_circ_0072387	OSCC	Up	–	Promote invasion and metastasis	(68)
Hsa_circ_0109291	OSCC	Up	–	Promote invasion and metastasis	(78)
CircRPMS1	NPC	Up	miR-203, miR-31和miR-451	Promote invasion and metastasis	(65)
CircCRIM1	NPC	Up	miR-422a/FOXQ1	Promote invasion and metastasis	(102)
CircCORO1C	LSCC	Up	let-7c-5p/PBX3	Promote invasion and metastasis	(77)	
CircHIPK3	NPC	Up	miR-4288/ELF3	Promote invasion and metastasis	(48)
Circ-ZNF609	NPC	Up	microRNA-150-5p/Sp1	Promote invasion and metastasis	(66)
CircCTDP1	NPC	Up	microRNA-320b/HOXA10/TGFβ2	Promote invasion and metastasis	(70)
Circ_0008450	NPC	Up	miR-577/CXCL9	Promote invasion and metastasis	(71)
CircARHGAP12	NPC	Up	EZR	Promote invasion and metastasis	(101)
Circ_0067934	LSCC	Up	miR-1324	Promote invasion and metastasis	(64)
CircFLNA	LSCC	Up	miR-486-3p/FLNA	Promote invasion and metastasis	(99)
CircRASSF2	LSCC	Up	miR-302b-3p/IGF-1R	Promote invasion and metastasis	(38)
CDR1as	LSCC	Up	miR-7/CCNE1 and PIK3CD	Promote invasion and metastasis	(17)
Hsa_circ_0023028	LSCC	Up	miR-194-5p	Promote invasion and metastasis	(69)
CircRNA_100290	LSCC	Up	miR-136-5p/RAP2C	Promote invasion and metastasis	(51)
Hsa_circ_0057481	LSCC	Up	miR-200c/ZEB1	Promote invasion and metastasis	(73)
CircMATR3	HSCC	Up	–	Promote invasion and metastasis	(72)
CircRNA_002178	OSCC	Up	Akt/mTOR	Promote invasion and metastasis	(42)
Hsa_circ_0066755	NPC	Up	miR-651	Promote invasion and metastasis	(80)
Hsa_circ_0004491	OSCC	Down	EMT related protein	Inhibit invasion and metastasis	(37)
circRNA_0000140	OSCC	Down	miR-31/LATS2	Inhibit proliferation	(50)
Hsa_circRNA_100533	OSCC	Down	miR-933/GNAS	Inhibit invasion and metastasis	(62)
Hsa_circ_0007059	OSCC	Down	AKT/mTOR	Inhibit invasion and metastasis	(41)
Hsa_circ_0055538	OSCC	Down	p53/Bcl-2/caspase	Inhibit invasion and metastasis	(53)
Circ-PKD2	OSCC	Down	miR-204-3p/APC2	Inhibit invasion and metastasis	(81)
Hsa_circ_0002203	OSCC	Down	–	Inhibit invasion and metastasis	(79)
CircLARP4	NPC	Down	ROCK1	Inhibit invasion and metastasis	(60)
Hsa_circ_0042666	LSCC	Down	miR-223/TGFBR3	Inhibit invasion and metastasis	(61)

The PI3K/Akt/mTOR, IGF-1/IGF-1R, Hippo, P53, and TGFβ signaling pathways mentioned above also play a role in the regulation of HNC invasion and metastasis by circRNA (38, 40–42, 45, 50, 75, 104). For example, TGF-β can act on the transcription factors Snail, Twist, etc., and is a key factor in inducing epithelial mesenchymal transition (EMT) (105). EMT is an important biological process for malignant tumor cells derived from epithelial cells to acquire the ability of migration and invasion (105). As mentioned above, circUHRF1 competitively binds miR-526b-5 and positively regulates c-Myc, which activates the TGF-β1 pathway (45). Therefore, circUHRF1 can promote the EMT and invasion and metastasis of HNC by activating the TGF-β1 pathway.

ZEB1 is also an important factor which promotes tumor EMT (106). It can work with snail and slug to inhibit downstream adhesion molecules and promoter molecules, and ultimately induce EMT. Hsa_circ_0057481 can directly capture miR-200c, promote the expression of the ZEB1, and promote the invasion and metastasis of LSCC (73).

Matrix metalloproteinases (MMPs) can promote tumor invasion and metastasis by promoting the degradation of the extracellular matrix (107). Hsa_circ_0001162 is a circRNA derived from MMP9, which blocks the inhibitory effect of miR-149 on the 3′ end of MMP9 by adsorbing miR-149, and promotes the migration and invasion of OSCC (100). In addition, there is also an AU-rich element RBP 1 binding motif on MMP9 mRNA 3′-UTR, which does not overlap with the binding site of miR-149 on MMP9 3′-UTR. Therefore, hsa_circ_0001162 can also enhance the stability of MMP9 mRNA by antagonizing the attenuation of MMP9 mRNA induced by the AU-rich element RBP 1 in OSCC (100). Rap2c is a member of the RAS oncogene family and it is reported that it can increase the activity of MMP2. Highly-expressed circRNA_100290 promotes the migration of LSCC by blocking miR-136-5p inhibiting this protein (51).

The realization of tumor migration and invasion requires the rearrangement of intracellular cytoskeletal proteins. The protein encoded by the EZR gene is a component of the microvilli, acting as an intermediate product between the plasma membrane and the actin cytoskeleton, and can regulate cell adhesion and skeletal reorganization (108). Studies have found that circARHGAP12 can bind to the 3′-UTR of EZR mRNA and promote its stability (101). EZR can interact with TPM3 and RhoA to regulate cytoskeleton remodeling and promote NPC invasion and metastasis (101). Rock1 protein plays a key role in cell surface structure adhesion and cytoskeleton recombination (109). Pan et al. proved that ROCK1 is the direct target of circLARP4, which inhibits cell migration and invasion of NPC by regulating the kinase (60). FLNA can limit tumor migration by regulating the activation of integrins and the morphology and degradation of focal adhesions (110). CircFLNA can act as a miRNA sponge for miR-486-3p, thereby protecting FLNA from miR-486-3p-mediated silencing, and inhibiting the migration of LSCC cells (99).

## CircRNAs Are Related to the Regulation of Tumor Glucose Metabolism in HNC

**Figure 3:**
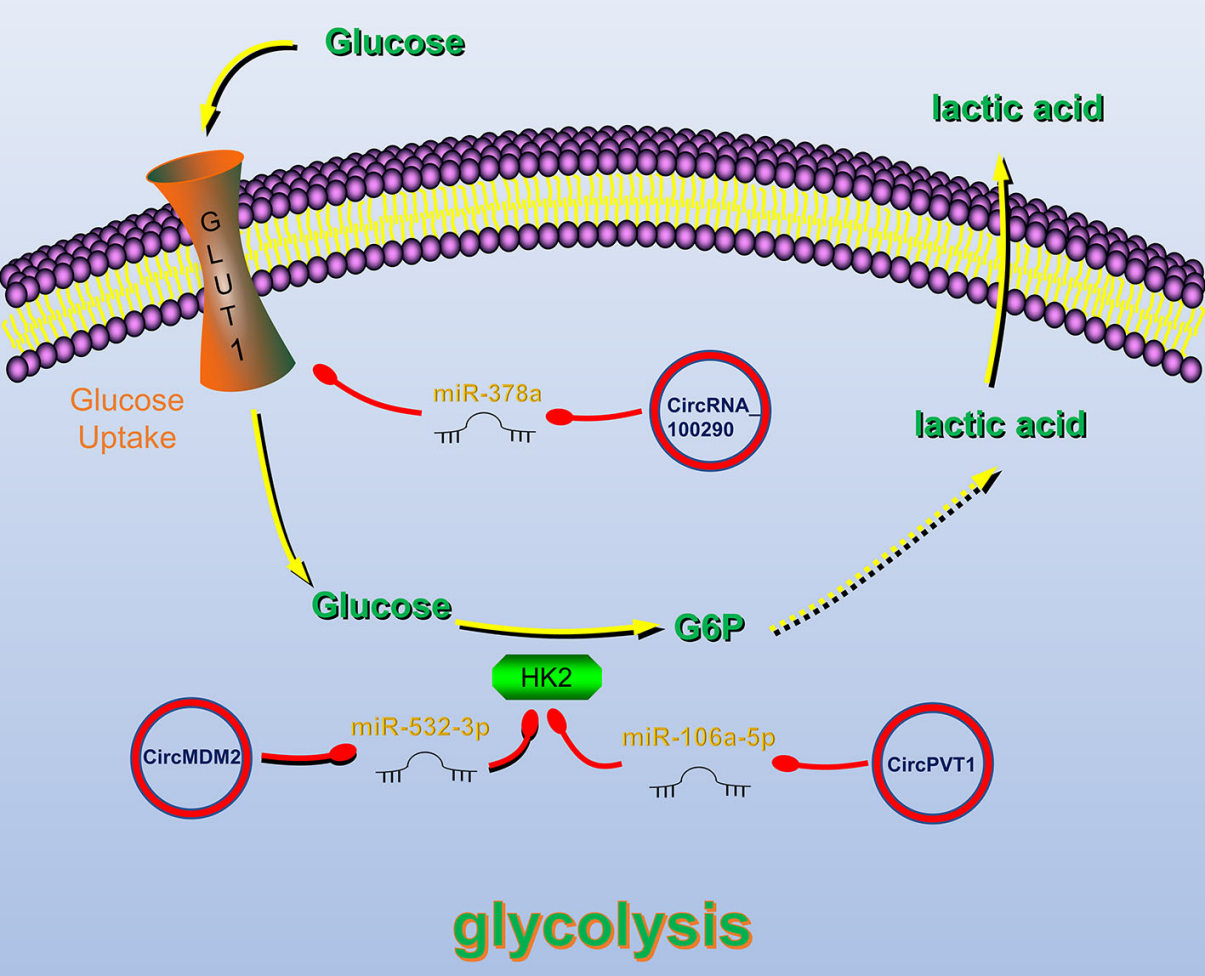
Circular RNAs (circRNAs) related to regulation of tumor glucose metabolism in head and neck cancers (HNC). CircRNAs that are known to regulate glucose metabolism processes and their validated targets in HNC. CircRNAs regulate HNC glucose metabolism by regulating Glucose transporter 1 (GLUT1) and Hexokinase 2, key enzymes of glycolysis. Red circle, upregulated circRNAs in HNC; green circle, downregulated circRNAs in HNC; yellow arrow, positive regulation; red arrow, negative regulation.

The various biological characteristics of tumors are closely related to the metabolism of substances and energy. Tumor metabolism provides energy for the biological behavior of tumors, promotes tumor growth, metastasis, and other activities (111). CircRNAs such as circPVT1 (112), circ_0000140 (113), circRNA_100290 (114), CDR1as (57), circMDM2 (63), hsa_circ_0072387 (68) and circGDI2 (115) (**Table 3**) can also affect tumor growth through glucose metabolism (**Figure 3**).

**Table 3:** The glycolysis-related circular RNAs in head and neck cancers.

CircRNAs	Primary sites of cancer	Expression change	Mechanism	Functions	References
CircMDM2	OSCC	Up	miR-532-3p/HK2	Promote glycolysis	(63)
CircRNA_100290	OSCC	Up	miR-378a/GLUT1	Promote glycolysis	(114)
CircPVT1	OSCC	Up	miR-106a-5p/HK2	Promote glycolysis	(112)
hsa_circ_0072387	OSCC	Up	–	Promote glycolysis	(68)
CDR1as	NPC	Up	miR-7-5p/E2F3	Promote glucose metabolism	(57)
Circ_0000140	OSCC	Down	miR-182-3p/CDC73	Inhibit glycolysis	(113)
CircGDI2	OSCC	Down	miR-424-5p/SCAI	Inhibit glycolysis	(115)

Glucose transporter 1 (GLUT1) plays an important role in the glycolysis process by regulating glucose uptake (116). CircRNA_100290 can alleviate the inhibitory effect of miR-378a on GLUT1. Therefore, overexpression of circRNA_100290 can increase the glucose uptake rate of tumor cells and meet their high metabolic need (114). Hexokinase 2 (HK2) is also a key enzyme in the glycolysis pathway, and its expression in HNC cells is significantly increased (117). Studies have shown that circRNAs can regulate the HK2 gene, thereby affecting the glycolysis of HNC cells. For example, in OSCC, circPVT1 and circMDM2 upregulate HK2 expression by inhibiting miR-497-5p and miR-532-3p, respectively, thereby promoting glycolysis (63, 112).

## CircRNAs Are Associated With Radiochemotherapy Resistance in HNC

**Figure 4:**
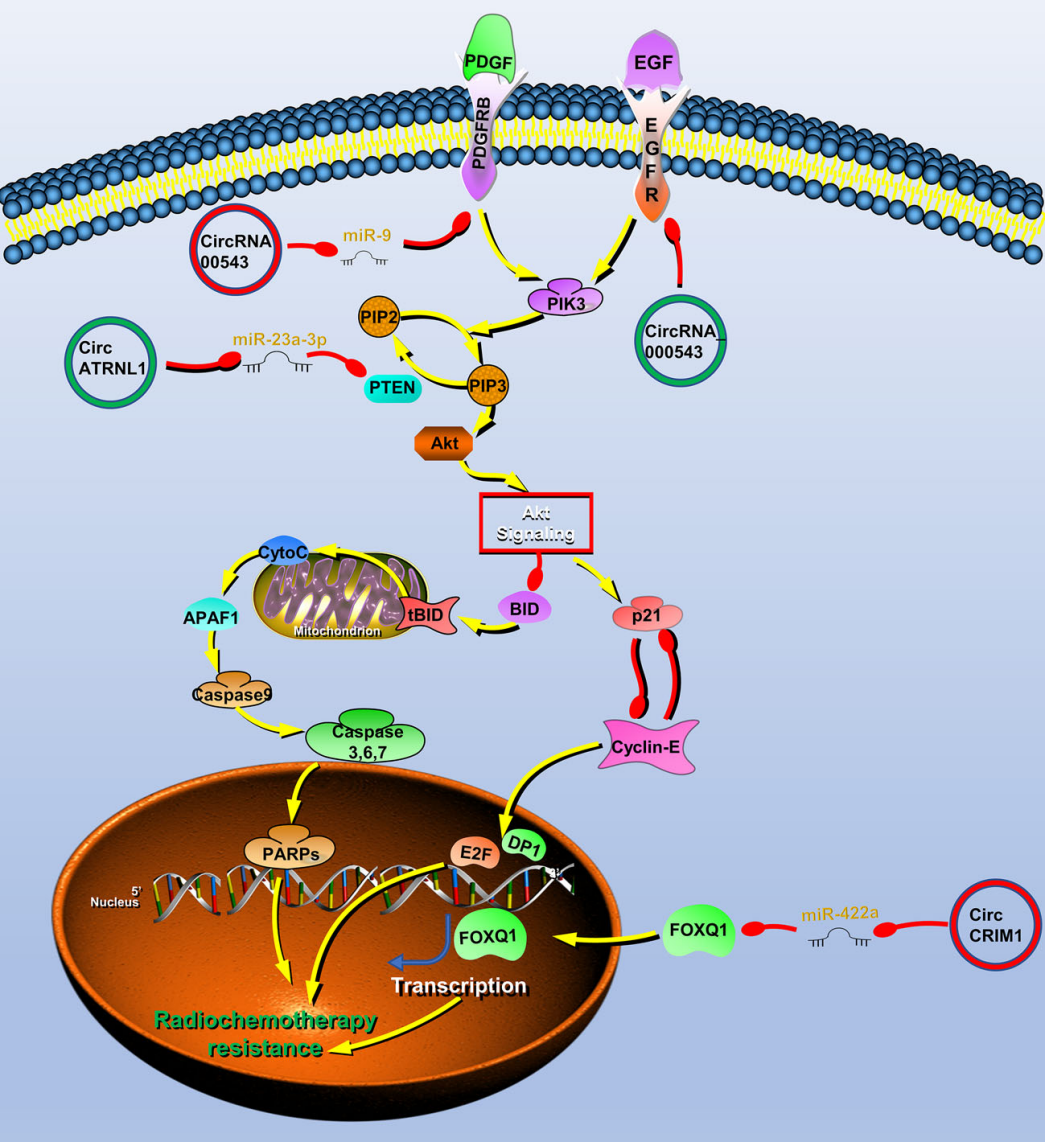
Circular RNAs (circRNAs) associated with radiochemotherapy resistance in head and neck cancers (HNC). CircRNAs that are known to regulate radiochemotherapy resistance processes and their validated targets in HNC. CircRNAs regulate the radiochemotherapy resistance of HNC through phosphatase and tensin homolog (PTEN)/PI3K/Akt/mTOR, epidermal growth factor receptor (EGFR), platelet-derived growth factor receptor A (PDGFR) signaling pathway and transcription factor forkhead box Q1 (FOXQ1). Red circle, upregulated circRNAs in HNC; green circle, downregulated circRNAs in HNC; yellow arrow, positive regulation; red arrow, negative regulation.

Surgery combined with radiotherapy and chemotherapy is the main treatment for HNC, but many patients develop radiochemotherapy resistance. Therefore, exploring the molecular mechanisms of HNC radiochemotherapy resistance has important practical significance for further improving the therapeutic effect. Recently, several lines of evidence have suggested that circRNAs, such as circRNA_000543 (118), circRNA_0000285 (119), hsa_circRNA_001387 (120), circATRNL1 (40) circ_0001971 (33), hsa_circ_0109291 (78), circCRIM1 (102), hsa_circ_0028007 (121), hg_circ_0005033 (122) and hsa_circ_0005379 (44) (**Table 4**) are likely to play vital roles in radiochemotherapy resistance in HNC (**Figure 4**).

**Table 4:** The radiochemotherapy resistance related circular RNAs in head and neck cancers.

CircRNAs	Primary sites of cancer	Expression change	Mechanism	Functions	References
Circ_0001971	OSCC	Up	miR-194 and miR-204	Inhibit chemotherapy sensitivity	(33)
Hsa_circ_0109291	OSCC	Up	–	Inhibit cisplatin sensitivity	(78)
CircRNA_000543	NPC	Up	miR-9/PDGFRB	Inhibit radiosensitivity	(118)
CircCRIM1	NPC	Up	miR-422a/FOXQ1	Inhibit docetaxel sensitivity	(102)
CircRNA_0000285	NPC	Up	–	Inhibit radiosensitivity	(119)
Hsa_circ_0028007	NPC	UP	–	Inhibit chemotherapy sensitivity	(121)
Hsa_circRNA_001387	NPC	UP	–	Inhibit radiosensitivity	(120)
Hg_circ_0005033	LSCC	Up	miR-4521	Inhibit chemotherapy sensitivity	(122)
Hsa_circ_0005379	OSCC	Down	EGFR	Promote cetuximab sensitivity	(44)
CircATRNL1	OSCC	Down	miR-23a3p/PTEN/PI3K/Akt	Promote radiosensitivity	(40)

To find radiotherapy resistance-related circRNAs, Chen et al. performed high-throughput sequencing on radiotherapy-sensitive and -insensitive NPC tissues and found that the expression of circRNA_000543 was upregulated in radiotherapy-insensitive NPC, which may be a carcinogenic circRNA involved in radiotherapy resistance. Further studies have shown that miR-9 can bind to circRNA_000543, and a bioinformatics software predicts that miR-9 can bind to the 3′ UTR of the PDFGRB. Therefore, the author finally proved that circRNA_000543 is involved in radiation resistance of NPC by blocking inhibition of miR-9 to PDFGRB (77).

Radiotherapy is also one of the main treatment methods of OSCC. The resistance to radiation often leads to poor prognosis and tumor recurrence in patients with OSCC. As mentioned above, circATRNL1 in OSCC induces tumor apoptosis and cell cycle arrest through PTEN/PI3K/AKT. Therefore, circATRNL1 also improves the radiosensitivity of OSCC cells through this mechanism (40).

Systemic induction chemotherapy is also an important auxiliary method. Recent studies reported that circCRIM1 can promote NPC metastasis and docetaxel chemoresistance. CircRNA sequencing was performed on high- and low-metastatic ability cell lines and obtained metastasis-related circRNA expression profiles, among which circCRIM1 was the most upregulated molecule. Further studies have shown that circCRIM1 upregulates FOXQ1 expression by acting as a miR-422a sponge, thereby promoting the process of NPC. The author performs enrichment analysis on the expression profile of NPC with high and low expression of FOXQ1 and found that chemoresistance was significantly rich. Furthermore, *in vitro* and *in vivo* experiments both showed that circCRIM1 promotes chemoresistance of NPC docetaxel (102). Cetuximab is an IgG1 monoclonal antibody with high affinity for the epidermal growth factor receptor (EGFR) (44). It can inhibit cell cycle progression and induce tumor cell apoptosis by specifically binding to the extracellular EGFR domain. Su et al. found that hsa_circ_0005379 can act on EGFR to significantly enhance the sensitivity of OSCC to cetuximab drugs (123). Functional experiments show that hsa_circ_0005379 can inhibit the growth and metastasis of OSCC, and increase the sensitivity to cetuximab drugs (123).

## CircRNAs Produced by Viruses in HNC

EBV and HPV are closely related to the pathogenesis of HNC. It is reported that about 98% of NPC patients are EBV positive (124), and about 70% of oral and pharyngeal squamous cell carcinoma are associated with HPV infection (125). Therefore, it is very important to understand the carcinogenic mechanism of the virus. Studies have shown that DNA viruses can produce circRNA in host cells, and these circRNAs may also be a potential mechanism of virus carcinogenesis (126, 127).

EBV is the first virus reported to produce circRNAs in the host genome. Toptan et al. used circRNA Seq to sequence the EBV genome and found that the BART, LMP2, and BHLF1 genes in the EBV genome can produce circRNA (126). In another study, the authors developed a tool called circleVis and found more than 30 circRNAs produced by EBV (127). Among them, circRPMS1 encoded by BART has been verified by other studies, and it has been found that circRPMS1 can promote the proliferation and metastasis of NPC by acting as a miRNA sponge (65, 128). circLMP2A was also verified to exist in gastric cancer, but there is no research of circLMP2A in HNC (129). Similarly, HPV can also produce circRNAs. Wang et al. found that the E7 gene in HPV16 virus can produce circular RNA-CircE7 in cervical cancer, and CircE7 can be translated into protein and promote tumor growth (130). However, the study of circRNAs produced by HPV in HNC has not been reported yet. Whether the circRNA produced by these tumor viruses also functions in HNC is worthy of further investigation.

## Conclusion and Future Prospects

CircRNAs have a closed-loop structure, lack free ends, and are not easily hydrolyzed by RNase. In addition, because of their high conservation, stability, expression abundance, and tissue and disease expression-specificity, circRNAs have the potential to become diagnostic or predictive biomarkers for disease. Notably, circRNAs can be detected in body fluids including peripheral blood, saliva, and urine. For example, there are a large number of circRNAs in human saliva (131), and hsa_circ_0001874 and hsa_circ_0001971 in saliva may be used as biomarkers for OSCC (33). In addition, circRNAs are enriched in exosomes (132). CircRASSF2 has been reported to be upregulated in serum exosomes in patients with LSCC and affects the progression of LSCC (38). These characteristics make the detection of circRNAs in body fluids of patients with HNC possible and have great clinical value and significance.

In HNC, circRNAs can affect tumor development through various mechanisms such as cell cycle, apoptosis, EMT, metabolism, and radiochemotherapy resistance. However, circRNAs also affect tumor development in other ways, such as immune escape, cancer stem cells. Immune checkpoint refers to a group of factors involved in negative regulation in the immune system and is closely related to the immune escape mechanism (133). Recently, inhibitors of immune checkpoint PD1 or PDL1 have been approved for the treatment of advanced HNC (134), and have become a new trend in the clinical treatment of HNC. Studies have shown that circRNA can also play a regulatory role in immune checkpoints, and may be used as a target for immune checkpoint therapy. For example, Circ-CPA4 acts as miRNA sponge for let-7 to down-regulate the expression of PD-L1, thereby promoting lung cancer cell death (135), while circFGFR1 can induce lung cancer cell resistance to PD-1 drugs by inhibiting miR-381-3p (136). However, the study of circRNA related to PD1 or PD-L1 in HNC has not been reported yet, and further research is needed. In addition, anti-tumor research for innate immunity is also very necessary. It has recently been reported that exogenous circRNA can activate innate immunity and is an effective adjuvant for inducing anti-tumor immunity *in vivo* (137). Exogenous viruses associated with HNC can also produce circRNA, such as EBV and HPV (65, 138–140). Therefore, the role of exogenous circRNA encoded by EBV or HPV genome in innate immunity deserves further study. In short, more efforts are needed to promote the research on the immune regulation of circRNA in HNC.

Cancer stem cells have the ability to induce tumor self-renewal and high chemoradiotherapy resistance in tumors, so cancer stem cells are one of the main reasons for drug resistance and high recurrence in tumor treatment (141). In HNC, circRNA participates in chemoradiotherapy resistance by regulating cancer stem cells. For example, curcumin mediates the regulation of cancer stem cell-like cells through the circRNA network and enhances the radiosensitization effect of NPC (142). In addition, Hg19_circ_0005033 affects LSCC stem cells, promotes tumor occurrence and chemotherapy resistance (122). However, the research on cancer stem cells in HNC just emerges, and there may be other regulatory mechanisms for circRNA. The main pathways involved in cancer stem cells are Hedgehog, Wnt, PI3K/PTEN, Notch, and Hippo signaling pathways (143), and many circRNAs in HNC are related to some of these signaling pathways (28, 82). Therefore, circRNAs may also participate in the regulation of cancer stem cells through these signaling pathways. Studying the role of circRNA in cancer stem cells will contribute to the improvement of tumor diagnosis and treatment methods, and further research is needed.

As with some studies, this manuscript has also limitations. In HNC, circRNAs affect tumor progression mainly by acting as miRNA sponges or binding proteins. But a few circRNAs with open reading frames can translate functional proteins under the mediation of IRES or m6A. Since there is no report on circRNA encoded protein in HNC, this article does not cover it. However, it cannot be ruled out that the circRNA in HNC can also play a role through the mechanism of encoding protein. In addition, the current research on circRNA has just emerged, and more clinical research is needed before clinical application. However, with the development of research, circRNAs will play a more important role. The emergence of circRNAs has greatly enriched the regulatory network of non-coding RNAs in human diseases and which may be used as new disease biomarkers and therapeutic targets with great potential.

## Author Contributions 

PL, KZ, YM, XD, XJ, LS, CG, WZ, ZZ, GL WX, SZ, and ZG conceived and wrote the manuscript. PL and ZG did the figure. All authors contributed to the article and approved the submitted version.

## Funding 

The present study was supported by the National Natural Science Foundation of China (grant nos. 81803025 and 81772901).

## Conflict of Interest

The authors declare that the research was conducted in the absence of any commercial or financial relationships that could be construed as a potential conflict of interest.

The reviewer YW declared a shared affiliation, with no collaboration, with the authors to the handling editor at the time of the review.
